# Elevated Expression of Selenoprotein Synthesis Enzyme SEPHS2 Driven by ATF4 Promotes the Resistance to Ferroptosis in Prostate Cancer

**DOI:** 10.1002/mco2.70908

**Published:** 2026-08-09

**Authors:** Shijia Bao, Xinxing Du, Zhenkeke Tao, Huixiang Xiao, Jia Zhang, Zhenzhen Wen, Wei‐Qiang Gao, Baijun Dong, Yu‐Xiang Fang, Jianjun Sha

**Affiliations:** ^1^ State Key Laboratory of Systems Medicine For Cancer Ren Ji Hospital Shanghai Cancer Institute Shanghai Jiao Tong University School of Medicine Shanghai China; ^2^ Department of Urology Ren Ji Hospital School of Medicine Shanghai Jiao Tong University Shanghai China; ^3^ Department of Urology Shanghai General Hospital Shanghai Jiao Tong University School of Medicine Shanghai China; ^4^ School of Biomedical Engineering & Med‐X Research Institute Shanghai Jiao Tong University Shanghai China; ^5^ Shanghai Key Laboratory of Cancer System Regulation and Clinical Translation Shanghai China

**Keywords:** activating transcription factor 4, ferroptosis, prostate cancer, selenophosphate synthetase 2

## Abstract

While it has been reported that antioxidant selenoproteins are crucial for anti‐ferroptosis in tumors, it remains unclear whether and how biosynthetic dysregulation of selenoproteins promotes resistance to ferroptosis. By bioinformatics analysis and in vitro confirmatory assays, we screened and identified selenophosphate synthetase 2 (SEPHS2), a key enzyme in a selenoprotein synthesis cascade, as a potential candidate for promoting anti‐ferroptosis in tumors. We found that the expression of SEPHS2 was upregulated upon treatment with ferroptosis inducers. Knockdown of SEPHS2 inhibited tumor proliferation, migration and invasion, as well as attenuated selenoprotein biosynthesis to sensitize tumor cells to ferroptosis. Furthermore, our results demonstrated that activating transcription factor 4 (ATF4), a ferroptosis‑related transcription factor, might work as a direct upstream regulator to activate SEPHS2 transcription. Downregulation of ATF4 enhanced sensitivity to ferroptosis, whereas restoration of SEPHS2 expression rescued this effect, suggesting that ferroptosis resistance might be regulated by the ATF4/SEPHS2 axis. Notably, for the in vivo assay, knockdown of either SEPHS2 or ATF4 exhibited the optimal repression of tumor growth when combined with ferroptosis inducers. Thus, our findings highlight that inhibition of ATF4/SEPHS2 signaling might contribute as a novel adjuvant therapeutic strategy to improve the efficacy of ferroptosis‐based treatment in tumors.

## Graphical abstract

Under the treatment of ferroptosis inducers, the expression of SEPHS2 is upregulated, driven by ATF4, to increase the biosynthesis of selenoproteins (e.g., GPX4) to promote resistance to ferroptosis.

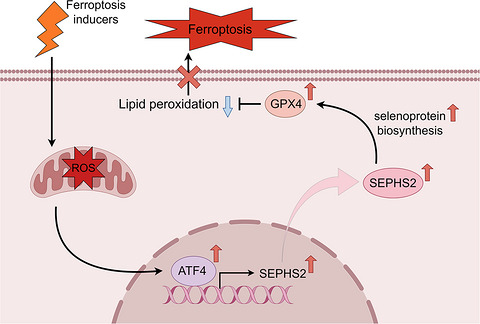

## 1 Introduction

Ferroptosis, a regulated cell death pathway, executes its lethal effects through an iron‐catalyzed process that drives lipid peroxide formation and deposition within the lipid bilayer of the cell membrane [1]. Previous studies have indicated that the intracellular antioxidant function of selenoproteins is tightly involved in the resistance to ferroptosis [2, 3]. However, how tumor cells regulate the synthesis or metabolism of selenoproteins to promote the resistance to ferroptosis remains unclear.

It is known that selenium‐derived selenocysteine is commonly found in the catalytic domain of multiple antioxidant selenoproteins [4], including glutathione peroxidases (GPXs) and thioredoxin reductases (TXNRDs). Several studies have provided evidence that these enzymes are essential for regulating intracellular redox balance, especially in tumor cells [5, 6, 7, 8]. Inhibiting their expression might be a promising adjuvant therapy for promoting ferroptosis in tumors. For example, inhibiting the expression of glutathione peroxidase 4 (GPX4), a primary ferroptosis regulatory selenoprotein [9], significantly enhances the sensitivity of tumor cells to the ferroptosis inducer [10]. Also, silencing selenoprotein thioredoxin reductase 1 (TXNRD1) would enhance the cytotoxicity of lysine oxidase, which could be suppressed by ferroptosis inhibitors in breast cancer [11]. On the other hand, the deficiency of selenium intake has also been indicated to induce reactive oxygen species (ROS) production and cell death [12, 13, 14]. However, it is still limited to know whether and how the ferroptosis inducer would promote the expression and/or the activity of selenoprotein synthesis‐related enzymes for their function as a resistance to ferroptosis.

In addition, transcriptional regulation has also been reported as a pivotal mediator in the modulation of ferroptosis sensitivity. For example, multiple transcription factors, including nuclear factor erythroid 2‐related factor 2 (NRF2) [15] and hypoxia‐inducible factor 1 subunit alpha (HIF‐1α) [16], were identified to be upregulated for resistance to ferroptosis via inhibition of lipid peroxidation. In addition, elevated expression of ATF4 promoted ferroptosis resistance in hepatocellular carcinoma (HCC) [17] but enhanced the sensitivity to ferroptosis inducers in triple‐negative breast cancer (TNBC) [18]. Therefore, these previous studies indicated a complex and tumor type‐related function of transcription factors in the regulation of ferroptosis.

In this study, we focused on selenophosphate synthetase 2 (SEPHS2), a crucial enzyme in phosphorylating hydrogen selenide (H_2_Se), which is a toxic precursor essential for the subsequent conversion steps in the synthesis of selenoproteins, including not only SEPHS2 itself [19] but also selenophosphate synthetase 1 (SEPHS1), GPXs, and TXNRDs [20, 21, 22]. We provide original evidence to uncover the important role of SEPHS2 in resistance to ferroptosis in prostate cancer by promoting the synthesis of selenoproteins. We also demonstrated that ferroptosis inducers could induce the activation of ferroptosis‐related transcription factor ATF4, thereby promoting the transcription of its downstream target gene SEPHS2.

## 2 Results

### 2.1 The Upregulation of SEPHS2 Expression Is Induced by the Treatment With Ferroptosis Inducers

**FIGURE 1:**
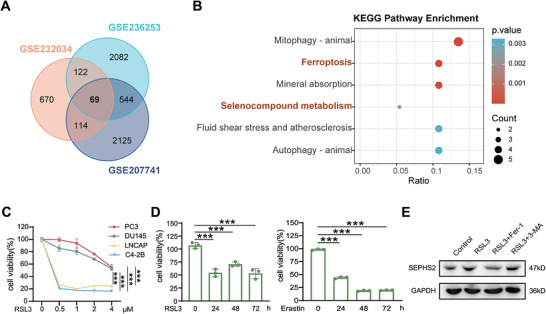
SEPHS2 expression is upregulated in response to ferroptosis inducers. (A) The intersection of upregulating genes after treatment of ferroptosis inducers in different datasets. (B) KEGG pathways enriched by shared genes from (A). (C) Cell viability of PC‐3, DU145, LNCaP, and C4‐2B cells treated with gradient concentrations of RSL3 for 24 h. (D) Cell viability of LNCaP cells treated with RSL3 (0.2 µM) or Erastin (40 µM) for 0–72 h. (E) Western blot analysis of SEPHS2 expression in LNCaP cells treated for 72 h with RSL3 alone or combined with ferrostatin‐1 (Fer‐1) or 3‐methyladenine (3‐MA). Values represent the mean ± SD (*n* = 3 per group). Statistical analysis was performed using two‐way ANOVA followed by Tukey's test in (C), and one‐way ANOVA followed by Dunnett's test in (D).

In order to screen and identify potential selenoprotein synthesis‐related genes that might be involved in the resistance to ferroptosis in prostate cancer, we performed bioinformatics assays using ferroptosis‐treatment‐related public datasets from the GEO database. We screened differentially expressed genes (DEGs) from three independent datasets: GSE236253 (glioma cells with Erastin treatment [23]), GSE207741 (pancreatic cancer cells with RSL3 treatment [24]), and GSE232034 (prostate cancer cells with Erastin treatment [25]). After that, we intersected all the ferroptosis‐related candidates that exhibited either a significant upregulated (*n* = 69) or a significant downregulated (*n* = 253) expression compared to the control after treatment with ferroptosis inducers (Figure 1A and Table S4). Because of the general consensus that the treatment with ferroptosis inducers could upregulate the expression of genes related to ferroptosis resistance [26], we herein focused our attention on the above screened candidates with the elevated expression after the treatment with either Erastin or RSL3. By Kyoto Encyclopedia of Genes and Genomes (KEGG) pathway enrichment assay, we found that 15 of 69 upregulated candidates were recruited into six pathways with significant difference, including selenocompound‐metabolism‐related pathway as well as ferroptosis‐related pathway as a positive control (Figure 1B). Notably, only two selenoprotein‐synthesis‐related genes, SEPHS2 and TXNRD1, were enriched in the selenocompound‐metabolism‐related pathway (Figure 1B, Figure S1A,B, and Table S5). Given that SEPHS2 participates in the synthesis of TXNRD1 [27], we wondered whether SEPHS2 might be a potential ferroptosis‐inducible gene for promoting resistance to ferroptosis in tumors by regulating selenoprotein biosynthesis.

For further confirmation, we evaluated resistance to ferroptosis in LNCaP, C4‐2B, DU145, and PC‐3 cells and checked the endogenous expression of SEPHS2 in these cells. We found that high endogenous expression of SEPHS2 in PC‐3 and DU145 was positively correlated with relatively high cell viability after treatment with RSL3 or Erastin (Figure 1C and Figure S1C,D). Notably, by the long‐term observation, the endogenous expression of SEPHS2 in LNCaP cells was continuously upregulated along with the gradually increased cell viability during 3 days under the condition of RSL3 or Erastin treatment (Figure 1D and Figure S1E). Moreover, when the ferroptosis inhibitor ferrostatin‐1 (Fer‐1) but not inhibitors of other cell death pathways (e.g., 3‐methyladenine for autophagy) was co‐treated with RSL3 or Erastin, the expression of SEPHS2 was reversely repressed to a similar level to that of the control group (Figure 1E and Figure S1F). Taken together, these results indicated that expression of SEPHS2 could be induced to be upregulated as a response to ferroptosis and might be involved in the promotion of resistance to ferroptosis.

### 2.2 Knockdown of SEPHS2 Inhibits Tumor Malignancy and Enhances the Sensitivity of Tumor Cells to Ferroptosis

**FIGURE 2:**
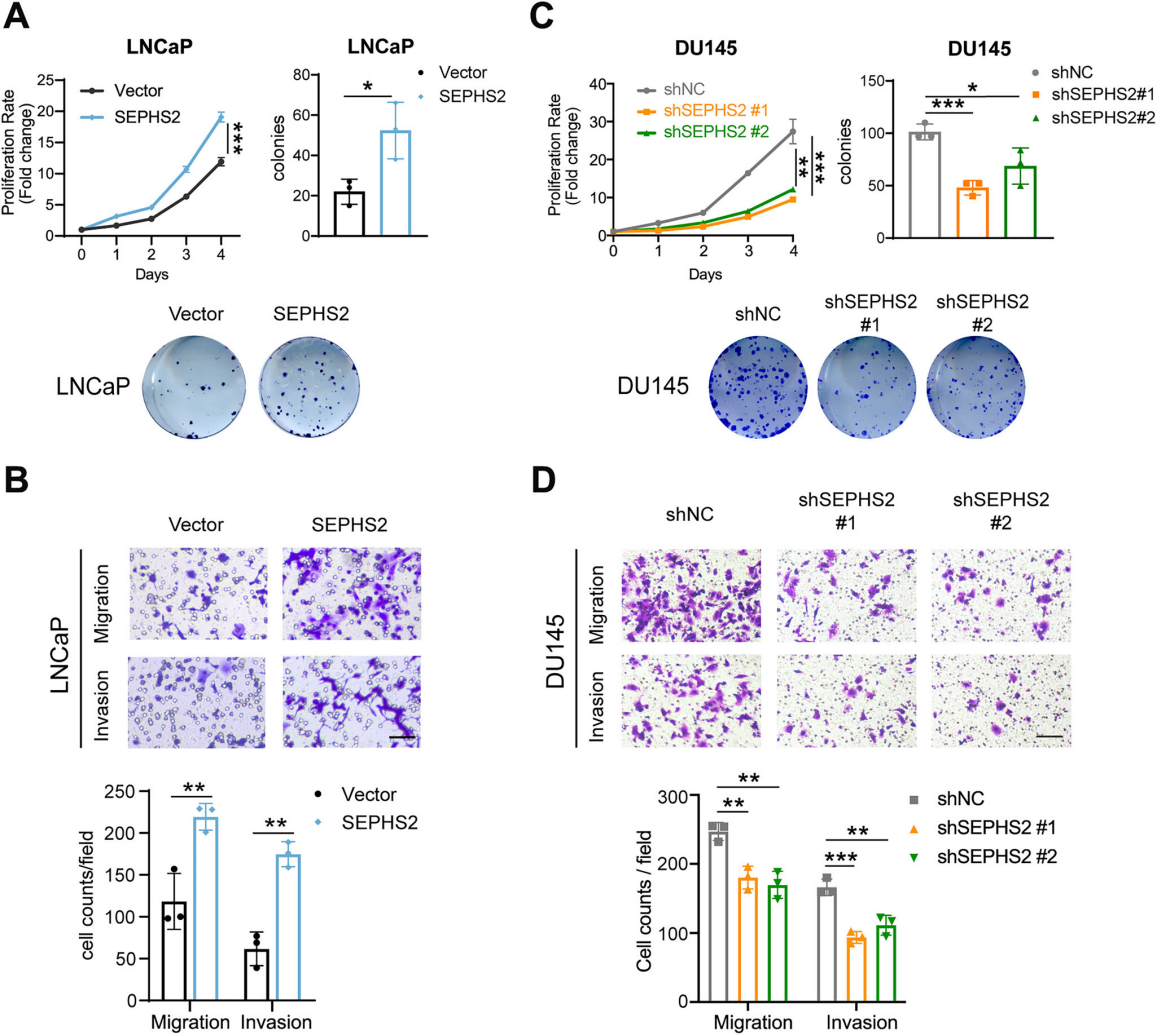
SEPHS2 knockdown inhibits while overexpression promotes tumor cell proliferation, migration, and invasion. (A) Cell proliferation assays in LNCaP cells with SEPHS2 overexpression or Vector control. (B) Representative images and quantification of migratory and invasive LNCaP cells with SEPHS2 overexpression or Vector control. Scale bar: 50 µm. (C) DU145 proliferation under SEPHS2 knockdown or non‐targeting shNC control. (D) Representative images and quantification of DU145 cell migration/invasion under SEPHS2 knockdown versus shNC. Scale bar: 50 µm. Values represent the mean ± SD (*n* = 3 per group). Statistical significance was analyzed by two‐way ANOVA and unpaired two‐tailed Student's *t*‐test in (A), unpaired two‐tailed Student's *t*‐test in (B), two‐way ANOVA with Tukey's test and one‐way ANOVA with Dunnett's test in (C), and one‐way ANOVA with Dunnett's test in (D).

We herein investigated whether SEPHS2 could regulate tumor malignance and, furthermore, whether this regulatory function was dependent on or independent of its potential anti‐ferroptosis ability. To this end, we carried out overexpression of SEPHS2 in LNCaP and C4‐2B cells but knockdown of it in both DU145 and PC‐3 cells, respectively, according to its endogenous expression level in these cells (Figure S2A,B). We found that overexpression of SEPHS2 in vitro could promote the proliferation, migration, and invasion in LNCaP and C4‐2B cells without treatment of ferroptosis inducers (Figure 2A,B and Figure S2C,D). As expected, inhibition of tumor cell proliferation as well as migration and invasion was observed after knockdown of SEPHS2 in either DU145 or PC‐3 cells in the absence of ferroptosis inducers (Figure 2C,D and Figure S3A,B). Consistently, SEPHS2 knockdown significantly attenuated metastasis in the orthotopic xenografts in nude mice (Figure S3C,D). These findings indicated that, besides resistance to ferroptosis, SEPHS2 might also participate in the regulation of tumor malignancy in prostate cancer.

**FIGURE 3:**
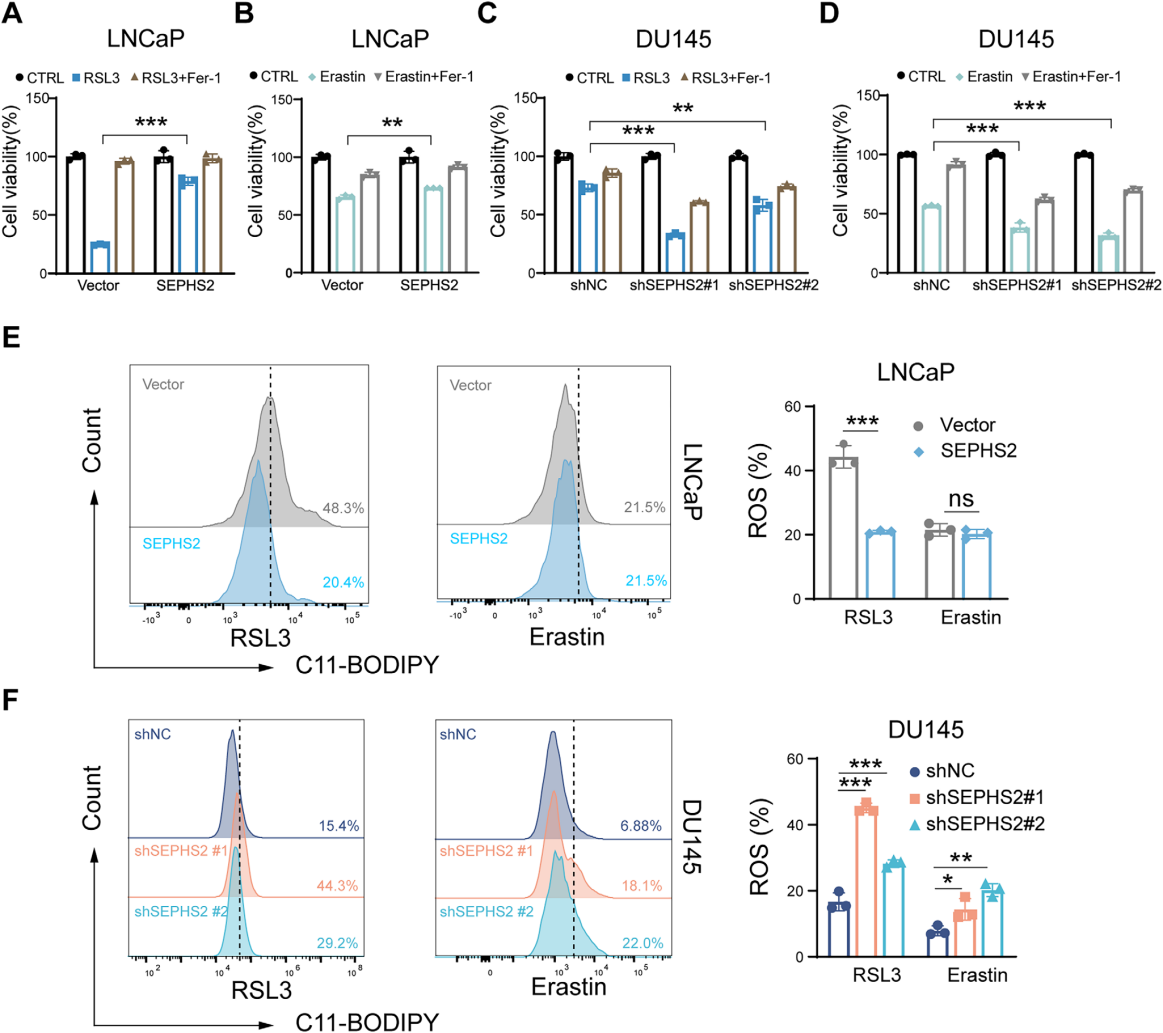
SEPHS2 knockdown sensitizes, whereas overexpression desensitizes tumor cells to ferroptosis. (A) Cell viability of LNCaP (vector vs. SEPHS2‐OE) cells treated for 24 h with RSL3 or RSL3 + Fer‐1. (B) Cell viability of LNCaP (vector vs. SEPHS2‐OE) cells treated for 24 h with Erastin or Erastin + Fer‐1. (C) Cell viability of DU145 (shNC vs. shSEPHS2) cells treated for 24 h with RSL3 or RSL3 + Fer‐1. (D) Cell viability of DU145 (shNC vs. shSEPHS2) cells treated for 24 h with Erastin or Erastin + Fer‐1. (E) Lipid ROS levels in LNCaP (vector vs. SEPHS2‐OE) cells treated for 24 h with RSL3 or Erastin. (F) Lipid ROS levels in DU145 (shNC vs. shSEPHS2) cells treated for 24 h with RSL3 or Erastin. Values represent the mean ± SD (*n* = 3 per group). Statistical significance was analyzed by unpaired two‐tailed Student's *t*‐test in (A–B) and (E), and one‐way ANOVA with Dunnett's test in (C–D) and (F).

**FIGURE 4:**
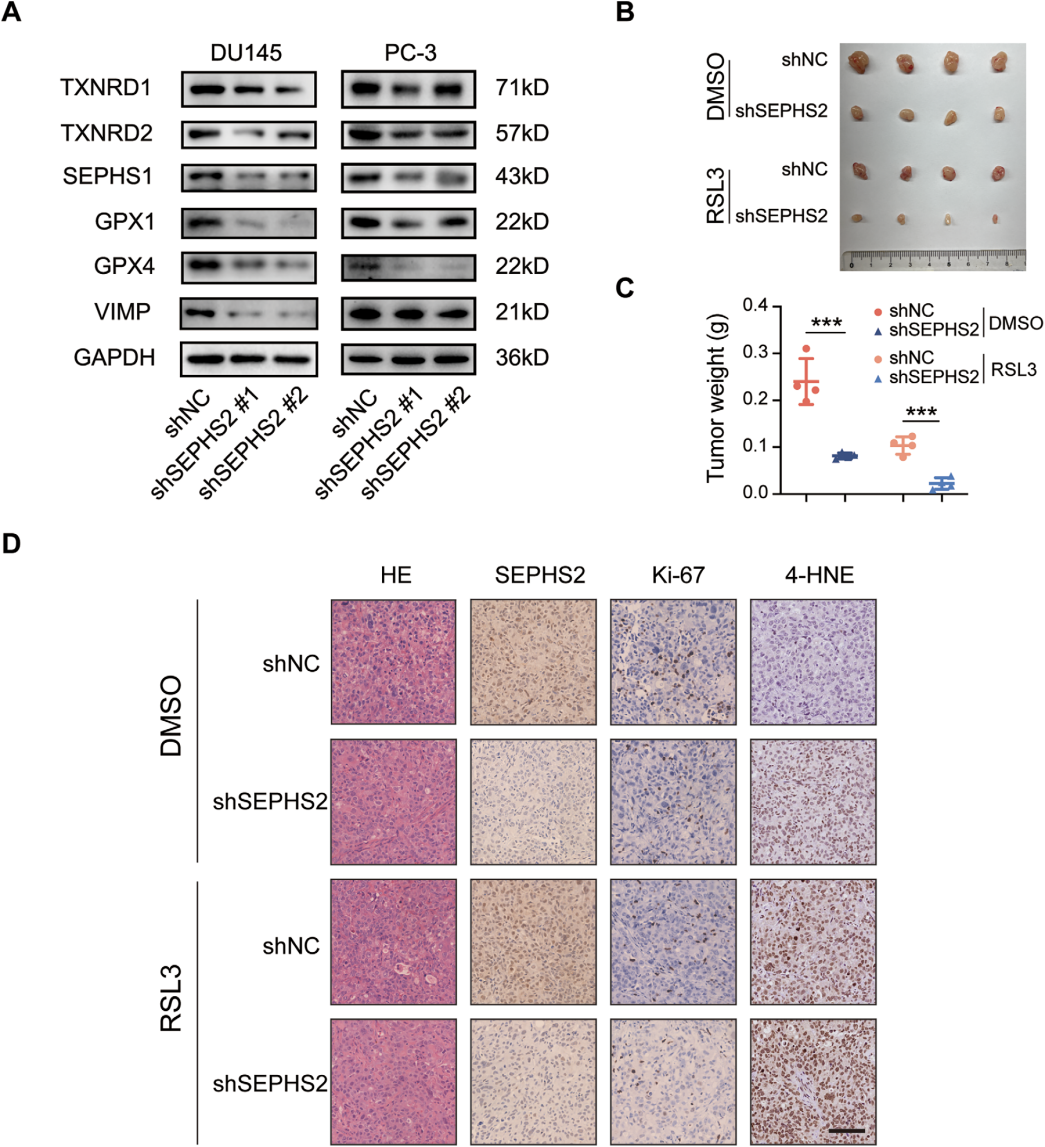
SEPHS2 knockdown suppresses selenoprotein expression and tumor growth. (A) Expression of selenoproteins in DU145 and PC‐3 cells with SEPHS2 knockdown. (B) Representative tumor images of DU‐145 xenografts with SEPHS2 knockdown (shNC vs. shSEPHS2) ± RSL3 treatment. (C) Weight analysis of DU‐145 xenografts with SEPHS2 knockdown (shNC vs. shSEPHS2) ± RSL3 treatment. (D) Representative IHC staining of SEPHS2, Ki67, and 4‐HNE in tissues from the xenografts. Scale bar: 100 µm. Values represent the mean ± SD (*n* = 4 per group). Statistical significance was analyzed by unpaired two‐tailed Student's *t*‐test in (C).

Moreover, neither overexpression nor knockdown of SEPHS2 caused a significant difference in apoptosis in tumor cells in the inducer‐free condition (Figure S4). However, overexpression of SEPHS2 enhanced, but knockdown of it impaired the cell viability when RSL3 or Erastin was treated (Figure 3A–D and Figure S5A). Similarly, overexpression of SEPHS2 decreased the level of lipid reactive oxygen species (Lipid ROS), while knockdown of it exhibited a reversed effect (Figure 3E,F and Figure S5B). Additionally, as a response, the expression changes in SEPHS2's downstream targets, such as TXNRD1 and GPX4, showed a positive correlation with the expression of SEPHS2 (Figure 4A and Figure S5C). Furthermore, supplementation with sodium selenite (Na_2_SeO_3_), the upstream substrate for selenoprotein synthesis, restored ferroptosis resistance in SEPHS2‐knockdown cells, evidenced by the increase of cell viability and the upregulated expression of TXNRD1 and GPX4 (Figure S5D,E). These results provided support to our hypothesis that SEPHS2 exerts its anti‐ferroptosis effect through regulating the selenoprotein biosynthesis. Thus, our data indicated that elevated expression of SEPHS2 promoted both tumor malignancy and resistance to ferroptosis. Also, it seemed that knockdown of SEPHS2 might be a potential adjuvant treatment for enhancing the sensitivity of tumors to ferroptosis. To further evaluate the therapeutic effects of SEPHS2 knockdown in vivo, we constructed a subcutaneous xenograft model in nude mice by inoculation of DU145‐shSEPHS2 or the control subclone cell line. After the treatment with RSL3 or dimethyl sulfoxide (DMSO), we found that knockdown of SEPHS2 alone could inhibit tumor growth. Interestingly, combination with the RSL3 treatment exhibited an optimal effect on repression of tumor growth through promoting the sensitivity to ferroptosis (Figure 4B,C), which was evidenced by the reduced Ki67 expression and the increased 4‐HNE expression (Figure 4D). Taken together, our findings indicated that knockdown of SEPHS2 could not only inhibit tumor malignance but also promote the sensitivity to ferroptosis in vitro and in vivo.

### 2.3 The Expression of SEPHS2 Is Upregulated by the Ferroptosis‐Related Transcription Factor ATF4 as Its Upstream

Since it has been evidenced that transcription factors contribute as a key regulator for response to ferroptosis [28], we herein also attempted to explore the potential upstream transcriptional regulator of SEPHS2, which might be activated by the treatment with the ferroptosis inducers. For this purpose, we performed RNA sequencing (RNA‐seq) on DU145 cells, which were treated with RSL3 or DMSO as a control, and analyzed the expression change of those transcription factors with potential links to ferroptosis pathways [29]. The analysis revealed that the expression of tumor protein p53 (*TP53*), *ATF4*, and endothelial PAS domain protein 1 (*EPAS1*) was upregulated, while specificity protein 1 (*SP1*), yes‐associated protein 1 (*YAP1*), WW domain containing transcription regulator 1 (*WWTR1*), nuclear factor erythroid 2‐like 2 (*NFE2L2*), BTB and CNC homology 1 (*BACH1*), and hypoxia‐inducible factor 1 subunit alpha (*HIF1A*) were downregulated in RSL3‐treated groups compared to DMSO controls (Figure S6A and Table S6). However, although the most upregulated expression of TP53 was measured after the treatment with RSL3, the loss‐of‐function mutations in the TP53 gene was reported to occur in a frequency of over 50% in prostate cancer [30], which usually lead to the disruption of its canonical transcriptional activity. Thus, we paid our attention to ATF4, the second most upregulated transcription factor with known stress‐responsive function [31], as the candidate for further investigation (Figure S6A and Table S6). As an identification, we checked the expression of ATF4 after the treatment with either RSL3 or Erastin, along with or without the ferroptosis inhibitor. We found that ATF4 expression could also be induced to be upregulated by ferroptosis inducers and could be again reversely repressed to its original level by simultaneously using the ferroptosis inhibitor (Figure S6B). Moreover, since integrated stress response (ISR) could be triggered by ferroptosis inducer RSL3 [32] and ATF4 has been reported as a key effector of ISR, of which the expression could be regulated by eukaryotic translation initiation factor 2 alpha (eIF2α) kinase signaling [33], we herein wondered whether elevated expression of ATF4 was caused by RSL3‐induced ISR. To this end, we checked the expression of both eIF2α and phosphorylated eIF2α (p‐eIF2α), two main biomarkers of ISR, after the treatment of RSL3 by western blot. Our results revealed that RSL3 treatment elevated p‐eIF2α levels while the expression of total eIF2α protein was similar to the control, suggesting that ISR might be activated by RSL3 to induce ATF4 expression (Figure S7A). Thus, our findings indicated that ATF4 might be a potential upstream transcription factor for anti‐ferroptosis via regulation of SEPHS2.

**FIGURE 5:**
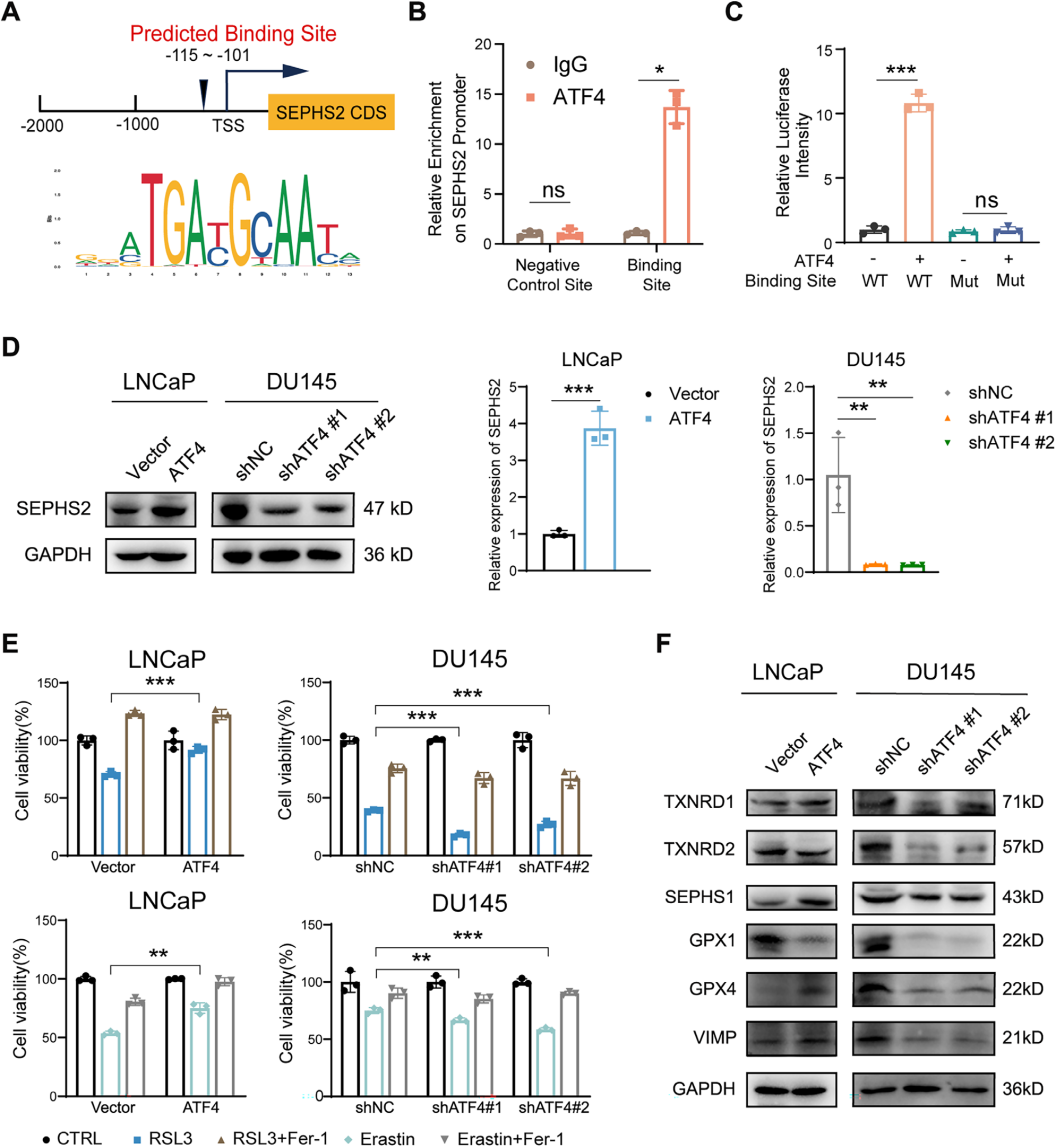
ATF4 controls ferroptosis sensitivity via SEPHS2 transcriptional regulation. (A) The predicted binding site and motif of ATF4 on the promoter region of SEPHS2. (B) ChIP‐qPCR assay of ATF4 binding on the promoter of SEPHS2 in DU145 cells. (C) Verification of ATF4 binding site on the promoter region of SEPSH2 through luciferase assay. (D) Western blot and qRT‐PCR analysis of SEPHS2 expression in ATF4‐OE LNCaP and ATF4‐knockdown DU145 cells. (E) Cell viability of LNCaP (vector vs. ATF4‐OE) and DU145 (shNC vs. shATF4) cells treated for 24 h with RSL3, Erastin, or RSL3/Erastin + Fer‐1. (F) Expression of selenoproteins in DU145 cells with ATF4 knockdown and in LNCaP cells with ATF4 overexpression. Values represent the mean ± SD (*n* = 3 per group). Statistical significance was analyzed by unpaired two‐tailed Student's *t*‐test in (B–C, D–E) and one‐way ANOVA with Dunnett's test in (D–E).

As expected, after we performed the related bioinformatic assay (Figure 5A) along with both chromatin immunoprecipitation‐quantitative polymerase chain reaction (ChIP‐qPCR) (Figure 5B) and luciferase reporter assay (Figure 5C), we predicted and identified a potential binding site of ATF4 but not TP53 (TTGTTGCACCTTTT) at the promoter region of *SEPHS2* (−115 ∼ −101 bp before the transcription start site), which indicated that ATF4 might be the upstream regulator of *SEPHS2* for anti‐ferroptosis by activating the transcription of *SEPHS2* (Figure 5A–C). In order to confirm our speculation, Next, we constructed relevant subclone cell lines in LNCaP cells for overexpression of ATF4 as well as in DU145 and PC‐3 cells for knockdown of it (Figure S7B). As expected, we observed that overexpression of ATF4 upregulated while knockdown of ATF4 downregulated the expression of SEPHS2 after the treatment with ferroptosis inducers (Figure 5D and Figure S7C). Consistent with the observation, the cell viability was significantly increased along with the enhancement of the expression of selenoproteins, including TXNRD1 and GPX4. In contrast, knockdown of ATF4 repressed the expression of selenoproteins so that the cell viability was impaired (Figure 5E,F and Figure S7D,E). On the other hand, since ferroptosis could also induce mitochondrial autophagy [34] and mitophagy related genes were enriched by our KEGG assay as well (Figure 1B), we interested whether ferroptosis induced upregulation of ATF4 was also involved in the regulation of mitophagy. To this purpose, we observed the ultrastructure of DU145 cells following RSL3 treatment by transmission electron microscopy (TEM). Our results revealed that typical mitophagy‐like structures, characterized by autophagic membranes engulfing swollen mitochondria with disrupted cristae, were exhibited in both shNC and shATF4 groups after 4 h of RSL3 exposure (Figure S7F). Also, Western blot analysis of the autophagy‐related proteins sequestosome 1 (p62) and microtubule‐associated protein 1 light chain 3 beta (LC3B), together with the mitochondrial marker translocase of outer mitochondrial membrane 20 (TOM20, also known as TOMM20), revealed a similar expression pattern between DU145 shATF4 and DU145 shNC cells following RSL3 treatment (Figure S7G). Taken together, these findings indicate that elevated expression of ATF4 contributed to the resistance to ferroptosis mainly via upregulation of SEPHS2 expression.

### 2.4 The Resistance to Ferroptosis Was Promoted by the ATF4/SEPHS2 Axis

**FIGURE 6:**
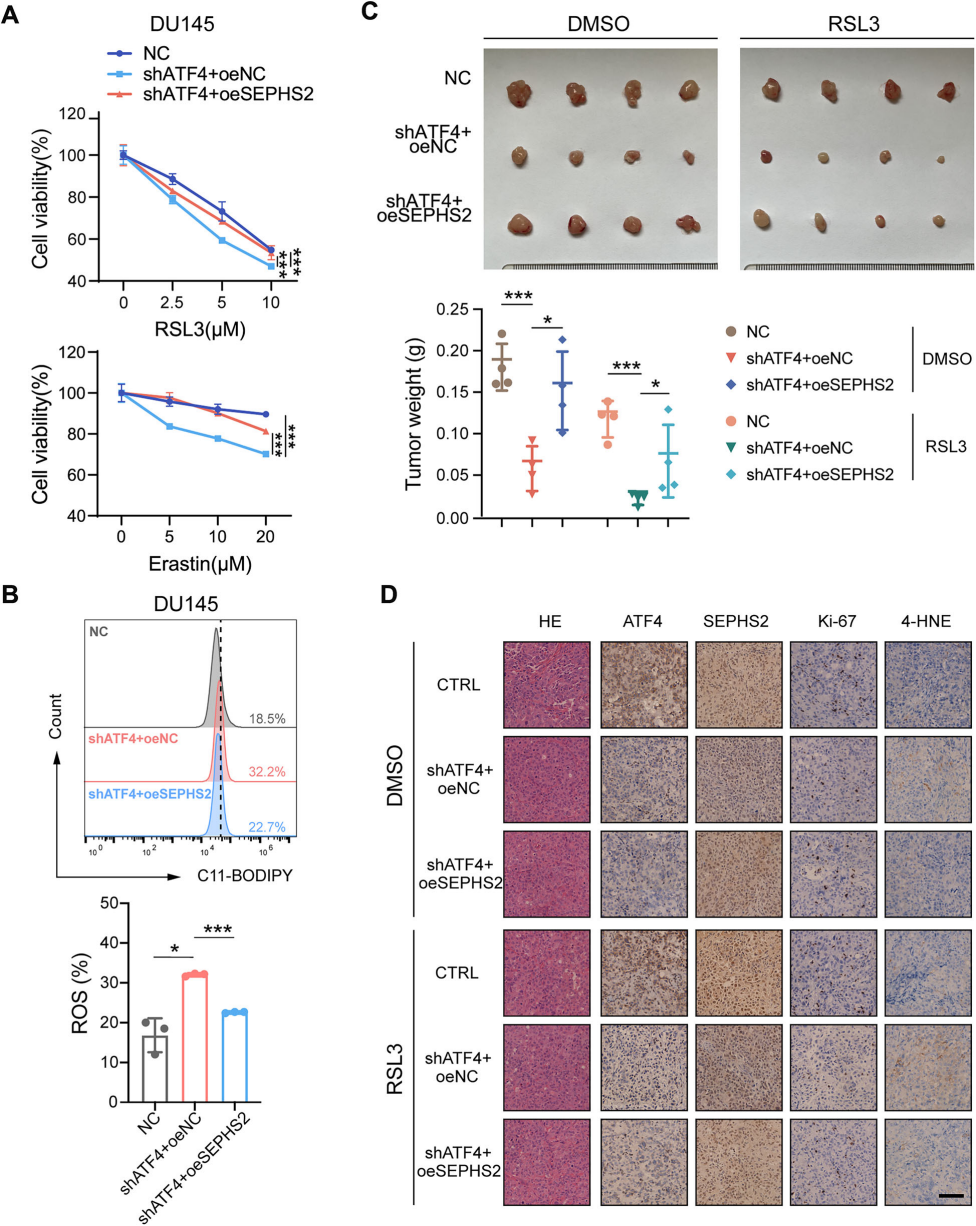
SEPHS2 overexpression can restore ferroptosis vulnerability caused by the knockdown of ATF4. (A) Assessment of cell viability in indicated cells following 24 h treatment with RSL3 or Erastin (*n* = 3 per group). (B) Analysis of lipid ROS in indicated cells following 24 h treatment with RSL3 (*n* = 3 per group). (C) Representative tumor images and weight analysis of indicated xenografts with or without RSL3 treatment (*n* = 4 per group). (D) Representative IHC staining of ATF4, SEPHS2, Ki67, and 4‐HNE in tissues from the xenografts. Scale bar: 100 µm. Values represent the mean ± SD. Statistical significance was analyzed by one‐way ANOVA with Dunnett's test.

Moreover, we further restored the expression of SEPHS2 after knockdown of ATF4 as a rescue to investigate whether the resistance to ferroptosis was promoted by the ATF4/SEPHS2 axis. We found that SEPHS2 overexpression restored the function of anti‐ferroptosis, while knockdown of ATF4 attenuated the resistance to ferroptosis by cell viability assay (Figure 6A and Figure S8A). Similarly, the lipid ROS assay also revealed a significant increase in ROS percentage by knockdown of ATF4. However, after restoration of SEPHS2 expression, the content of ROS was again reduced to a similar level to that in the control group (Figure 6B and Figure S8B). On the other hand, the expression of selenoproteins was repressed after knockdown of ATF4 but could be upregulated again when the expression of SEPHS2 was restored (Figure S8C). Furthermore, we constructed a relevant subcutaneous xenograft mouse model to evaluate whether blockage of the ATF4/SEPHS2 axis could effectively impair the resistance to ferroptosis and, in turn, inhibit tumor growth in vivo. Importantly, we found that tumor growth was significantly repressed after knockdown of ATF4. Moreover, when it was combined with the treatment with RSL3, the tumor wight was further reduced. In contrast, SEPHS2 overexpression exhibited the effect on restoration of tumor growth in certain degree regardless of the treatment with RSL3, mirroring our in vitro findings (Figure 6C). By immunohistochemistry (IHC) assay, the expression of SEPHS2 was observed to be repressed after knockdown of ATF4 along with the low expression of Ki67. Nevertheless, restoration of SEPHS2 expression upregulated Ki67 expression. When adding RSL3 as the adjuvant treatment to ATF4 knockdown, the lowest expression of Ki67 as well as the highest expression of 4‐hydroxy‐2‐nonenal (4‐HNE) were observed, which indicated that inhibition of ATF4 expression promoted the sensitivity to ferroptosis so to repress tumor growth. Additionally, when SEPHS2 was overexpressed as a rescue, the Ki67 expression was upregulated and the 4‐HNE expression was down regulated compared to that in the ATF4 knockdown group (Figure 6D). Collectively, these data indicated that ATF4 could be induced by ferroptosis to upregulate the expression of SEPHS2 to promote the resistance of tumor to ferroptosis in vitro and in vivo. Also, inhibition of the ATF4/SEPHS2 axis might be a promising adjuvant therapeutic strategy to restore the sensitivity to ferroptosis.

## 3 Discussion

While exogenous induction of ferroptosis is a promising new adjuvant therapy for tumors, the resistance to ferroptosis treatment is one of the main difficulties that needs to be overcome urgently in the process of clinical translation and application of this therapy. Although existing studies have pointed out the important regulatory role of selenoproteins in ferroptosis resistance [5], the regulatory mechanism for the expression changes of both selenoproteins and related genes, especially selenoprotein‐synthesis‐related genes, remains poorly understood. In this study, we screened and identified a key enzyme in selenoprotein synthesis, SEPHS2, whose upregulation can promote the resistance of tumor cells to ferroptosis. Additionally, we also found that its upstream transcription factor ATF4 can be induced and activated by ferroptosis inducers to upregulate the expression of SEPHS2. Our results suggest that blockage of the ATF4/SEPHS2 axis might be useful for enhancing the sensitivity of tumor cells to ferroptosis treatment.

As a key enzyme for selenium detoxification, SEPHS2 was found to be required for cancer cell survival in multiple cancers [35]. Although previous studies have indicated that elevated expression of SEPHS2 promotes tumor proliferation [36] and chemotherapy resistance [37], and is positively correlated with the invasiveness and malignant grade of tumors [38], the understanding of how SEPHS2 regulates the resistance of tumors to ferroptosis and the related molecular mechanism remains limited. In our study, we observed the elevated expression of SEPHS2 in multiple tumors, including prostate cancer. Mechanistically, we demonstrated that knockdown of SEPHS2 expression could effectively impair the resistance to ferroptosis in a selenoproteins biosynthesis‐dependent manner, which was evidenced by the increase of intracellular ROS levels, the attenuation of cell viability, and the downregulation of the expression of selenoproteins such as TXNRD1 and GPX4 as well. However, the labile nature and low abundance of selenoprotein biosynthetic intermediates, such as selenophosphate, limited reliable quantification of these metabolites and direct assessment of SEPHS2 enzymatic activity. Additionally, it remains to be investigated whether the observed ferroptosis sensitivity upon SEPHS2 knockdown could trigger unclear compensatory bypass mechanisms. Despite these limitations, our findings indicated a promising adjuvant therapeutic strategy to enhance the sensitivity of tumors to ferroptosis via inhibition of SEPHS2 expression.

It is worth mentioning that although peroxiredoxin 6 (PRDX6), a member of the thiol‐specific antioxidant protein family, was reported to be able to activate and interact with SEPHS2 to contribute to anti‐ferroptosis [39], the potential upstream regulatory mechanism of SEPHS2 expression still needs to be further investigated. Notably, in this study, we originally screened and identified a ferroptosis‐inducible transcription factor, ATF4, as a potential upstream of SEPHS2. Although it has been reported that a myeloblastosis oncogene (MYB)‐regulated acute myeloid leukemia (AML)‐enriched enhancer could promote SEPHS2 expression for AML survival [36], we found that the expression of ATF4 but not MYB exhibited a significant upregulation by RNA‐seq and western blot assay after the treatment with ferroptosis inducers. Moreover, when cells were co‐treated with the ferroptosis inhibitor Fer‐1 and ferroptosis inducers, ATF4 expression returned to a level similar to that of the control, indicating a ferroptosis inducible manner of its profiling change. Furthermore, RSL3‐induced upregulation of p‐eIF2α expression indicated that ferroptosis might induce elevated expression of ATF4 via triggering ISR. However, the detailed upstream mechanisms underlying the activation of ATF4 remained to be systematically elucidated.

Besides the oncogenic function of ATF4 in promoting tumor proliferation, metastasis, and chemotherapy resistance by upregulating the expression of its downstream target solute carrier family 7 member 11 (SLC7A11), activating the nuclear factor kappa B (NF‐κB) pathway, or targeting asparagine metabolism [40, 41, 42], our study provided several findings to support the hypothesis that upregulation of the ATF4/SEPHS2 axis, which activates selenoprotein biosynthesis, but not mitophagy, could promote resistance to ferroptosis. First, overexpression of ATF4 increased, but knockdown of it decreased tumor cell viability after the treatment with ferroptosis inducers both in vitro and in vivo. Second, ATF4 promoted the expression of SEPHS2 at the transcriptional level by directly recognizing and binding to the promoter region of SEPHS2. Third, restoration of SEPHS2 expression in the condition of ATF4 knockdown effectively attenuated the sensitivity of tumor cells to ferroptosis. Furthermore, the expression of those well‐reported selenoproteins was positively correlated with that of the ATF4/SEPHS2 axis. Consistently, the activation or inhibition of selenoproteins was observed along with the upregulation or downregulation of the axis, respectively, which was evidenced by the relevant decrease or increase in the level of intracellular lipid ROS. Taken together, contrary to previous reports, which indicated the function of ATF4 in anti‐ferroptosis by promoting SLC7A11 expression to maintain glutathione homeostasis and enhance the antioxidant defense capacity of tumor cells [17, 43], our study revealed a novel regulatory mechanism by which the resistance of tumor cells to ferroptosis is enhanced by upregulating the ATF4/SEPHS2 axis. However, whether other unknown downstream targets of ATF4 also participate in ferroptosis resistance in prostate cancer still needs to be investigated.

In summary, our study revealed a novel regulator for resistance to ferroptosis, SEPHS2, whose expression is elevated as a response to ferroptosis inducers to promote anti‐ferroptosis via activating selenoproteins biosynthesis. Moreover, our findings demonstrated a ferroptosis‐inducible transcription factor ATF4 as a potential upstream regulator of SEPHS2 that upregulates its expression. Thus, our study indicated that blockage of the ATF4/SEPHS2 axis might be a promising adjuvant therapeutic strategy for enhancing the sensitivity of tumor cells to ferroptosis.

## 4 Materials and Methods

### 4.1 Cell Lines and Cell Culture

The human prostate cell lines LNCaP, C4‐2B, PC‐3, and DU145, along with the human embryonic kidney cell line HEK293T, were sourced from the Cell Bank of the Chinese Academy of Sciences (Shanghai, China). All cell cultures were maintained in Dulbecco's modified Eagle medium (DMEM; MeilunBio, MA0212) containing 10% fetal bovine serum (FBS; MeilunBio, PWL001) and 1% penicillin/streptomycin (Beyotime, ST488). Cells were incubated at 37°C and 5% CO_2_ humid atmosphere.

### 4.2 Plasmids Construction

Short hairpin RNAs (shRNAs) against SEPHS2 or ATF4 were cloned into the pLKO.1 plasmid (Addgene, 42516#). The amplicons encoding SEPHS2 and ATF4 were individually cloned into the pLenti‐CMV‐MCS‐Puro Vector (MiaoLingBio, 73582), and the 60th amino acid position in SEPHS2 coding sequence (CDS) was mutated from a terminator UGA to cysteine [35]. All used primer sequences are listed in Table S1.

### 4.3 Lentiviral Transfection

For lentivirus production, HEK293T cells were co‐transfected with the lentiviral plasmid and the packaging plasmids psPAX2 (Addgene, #12260) and pMD2.G (Addgene, #12259) at a mass ratio of 3:2:1 using PEI as the transfection reagent in serum‐free DMEM. Six hours post‐transfection, the culture medium was replaced with DMEM supplemented with 10% FBS. Note that 48 h later, the supernatant was centrifuged and filtered for cancer cell transfection at a confluency of 50% in six‐well plates. Empty plasmids were used as a control. After 24 h of infection, the medium was changed, and 1–2 µg/mL puromycin (Biosharp, BS111) was added to screen out positive infected cells for 2 weeks to establish stable knockdown or overexpression cell lines.

### 4.4 RNA‐seq Assay

RNA‐seq was performed using DU145 cells after treatment with RSL3 or DMSO, as described in our previous study [44]. Briefly, total RNA was isolated from the indicated cells using an RNA extraction kit (Fastagen, 220011) following the manufacturer's protocol. Sequencing libraries were constructed and subsequently subjected to paired‐end sequencing by Biomarker Tech (Beijing, China) on an Illumina NovaSeq 6000 platform. Raw sequencing data were processed and analyzed via the BMKCloud online platform (www.biocloud.net), from which normalized DEGs were derived.

### 4.5 RNA Extraction and qRT‐PCR Assay

Total RNA was extracted with the RNAfast200 kit (Fastagen, 220011) according to the manufacturer's instructions. The product was reverse transcribed into cDNA using HiScript II Q RT SuperMix (Vazyme, R223‐01), and then amplified using ChamQ Universal SYBR qPCR Master Mix (Vazyme, Q711‐02). The relative expression of the target gene was quantified by the 2^−ΔΔCt^ method and normalized by the expression of GAPDH. All used primer sequences are listed in Table S2.

### 4.6 Western Blotting

Cellular protein was lysed using RIPA lysis buffer (Beyotime, P0013B) with Protease Inhibitor Cocktail (Biosharp, BL612A). The concentration of the protein was evaluated by a BCA protein assay kit (Thermo Fisher, 23227). Proteins obtained were diluted to the same concentration, resolved on a 10% SDS‐PAGE gel, and transferred to 0.45‐µm PVDF membranes. After blocking the membrane using non‐fat milk for 1 h, the membrane was incubated with primary antibodies at 4°C overnight. The next day, corresponding HRP‐conjugated secondary antibodies were used to hybridize the primary antibodies, and then the proteins were detected using Immobilon Western HRP Substrate (Millipore, WBKLS) and BioRad VersaDoc 4000 imaging system. Antibodies used and dilution factor are listed in Table S3.

### 4.7 Cell Proliferation Assay

Cell Counting Kit‐8 (CCK‐8; Biosharp, BS350A) was used according to the manufacturer's instructions. Briefly, 2000 cells were plated per well in 96‐well plates and cultured for 5 days. At each measurement time point, the existing medium was replaced with a serum‐free DMEM mixture with 10% (v/v) CCK‐8 reagent. After a 2‐h incubation at 37°C, the absorbance was measured at 450 nm using a microplate reader, with proliferation rates calculated from optical density (OD) values normalized to Day 0 controls.

### 4.8 Cell Viability Assay

Cell viability under ferroptosis induction was evaluated using the CCK‐8 assay following the same protocol. Cells were seeded in 96‐well plates at 10,000 cells per well. After the cells adhered, they were treated with increasing concentrations of the ferroptosis inducers RSL3 (MCE, HY‐100218A) or Erastin (MCE, HY‐15763) to assess their resistance to these drugs. To verify ferroptosis‐specific cell death, parallel experiments were performed with co‐treatment of 1 µM ferroptosis inhibitor ferrostatin‐1 (Fer‐1; MCE, HY‐100579). The inducers were applied at concentrations ranging from 0 to 10 µM for RSL3 and from 0 to 40 µM for Erastin, across various experiments as required to achieve the desired cytotoxic effect in different cell lines. The specific concentrations used in each experiment are detailed in the corresponding figures. The treatment duration varied from 24 to 72 h across experiments, as indicated in the respective figures, prior to CCK‐8 measurement. Viability was expressed as a percentage of untreated controls after background subtraction.

### 4.9 Colony Formation Assay

To evaluate the colony‐forming ability of prostate cancer cells, the cells were seeded in 12‐well plates at 200 cells per well and cultured for 10–14 days until colonies were visible. Note that 4% paraformaldehyde (PFA) was used to fix the cells for 15 min at room temperature. Then the colonies were stained with a crystal violet staining solution (Beyotime, C0121) for 20 minutes and photographed.

### 4.10 Transwell Migration and Invasion Assay

Note that 8‐µm‐pole transwell chambers (Corning, 3422) were used to assess the migration and invasion ability of prostate cancer cells. A total of 5 × 10^4^ cells were suspended in serum‐free DMEM and added into the upper chamber, while 100 µL DMEM was added into the lower well. For the invasion assay, the chambers were precoated with 50 µL of Matrigel (Corning, 356234) that had been diluted 1:8 in serum‐free DMEM. After the Matrigel coagulated, a cell suspension was added to the chamber. Note that 36–48 h later, cells attached to the lower side of the membrane were fixed with 4% PFA for 15 min and were stained with 0.2% crystal violet. The stained cells were imaged with a Leica microscope and counted with ImageJ software (version 1.53k).

### 4.11 Lipid Peroxidation Assay

A BODIPY 581/591 C11 probe (Thermo Scientific, D3861) was used to detect lipid ROS. Briefly, LNCaP, C4‐2B, DU145, and PC‐3 cells were seeded in six‐well plates and treated with RSL3 (0.4 µM for LNCaP and C4‐2B; 2 µM for DU145 and PC‐3) or Erastin (4 µM for C4‐2B, and 40 µM for LNCaP, DU145 and PC‐3) for 24 h. Following the treatment, the cells were collected, incubated with 2 µM of the BODIPY probe at 37°C for 30 min, and then analyzed immediately using the FITC channel on a flow cytometer (BD Accuri C6 Plus).

### 4.12 Apoptosis Assay

For the apoptosis assay, Annexin V‐fluorescein isothiocyanate (Annexin V‐FITC; Biosharp, BL110A) and 7‐aminoactinomycin D (7‐AAD; Beyotime, C1053S) were used according to the manufacturers' instructions. In brief, 5 µL of Annexin V‐FITC and 5 µL of 7‐AAD were added to 100 µL of cell suspension. The cells were incubated for 20 min at room temperature under dark conditions. Then the fluorescence of samples was detected at the FITC and PerCP‐Cy5.5 channels. FlowJo (version 10.4) was used to evaluate the data.

### 4.13 Chromatin Immunoprecipitation‐qPCR Assay

ChIP was performed using the SimpleChIP Enzymatic Chromatin IP Kit (Cell Signaling Technology, #9003S) according to the manufacturer's instructions. Briefly, 2 × 10^7^ DU145 cells were cross‐linked with formaldehyde to fix protein–DNA interactions, nuclei were isolated, and chromatin was enzymatically digested to ∼150 bp fragments. After nuclear lysis, immunoprecipitations were carried out overnight at 4°C with rotation using an anti‑ATF4 antibody; species‑matched normal IgG was included as a negative control. Immune complexes were captured with Protein G magnetic beads. Beads were washed, chromatin was eluted, cross‐links were reversed, and DNA was purified. Enrichment of ATF4 at the target promoter was quantified by qPCR. Primer sequences are listed in Table S2.

### 4.14 Luciferase Assay

Note that 293T cells were seeded in a 12‐well plate at a 50% density, ready for reporter plasmids transfection. To assess the effect of ATF4 on SEPHS2 promoter activity, cells were co‐transfected with one of two effector plasmids (either the oeATF4 plasmid or the control empty vector), a firefly luciferase reporter plasmid (pGL3 containing either the wild‐type or mutant SEPHS2 promoter), and the pRL‐TK Renilla luciferase plasmid as an internal control. The plasmids were transfected at a mass ratio of 1000 ng (effector):1000 ng (firefly reporter):400 ng (Renilla control). Forty‐eight hours post‐transfection, the cells were lysed, and the fluorescence intensity was measured and normalized by Firefly&Renilla Dual Luciferase Reporter Assay Kit (MeilunBio, MA0518) according to the manuals.

### 4.15 Tumor Xenograft Experiment

All animal‐related experiments were scrutinized and approved by the Committee for Ethical Review of Research Involving Animal Subjects at Renji Hospital (approval number: RA‐2022‐191), and ethical standards were consistent with national and international standards. The ordering and feeding for BALB/c nude mice were entrusted to the Laboratory Animal Investigation Center of Renji Hospital in a specific‐pathogen‐free standard environment.

For the evaluation of the therapeutic effect of RSL3 in vivo, 6‐week‐old male nude mice were used (*n* = 4 per group) for subcutaneous xenograft transplantation. Note that 5 × 10^4^ cells were resuspended in a 50% Matrigel‐PBS solution and inoculated into the right flank of the nude mice. One week later, RSL3 was injected intraperitoneally at a dose of 20 mg/kg every 2 days for 3 weeks. The RSL3 solution formulation: 5% 80 mg/mL RSL3‐DMSO solution + 40% PEG 300 + 5% Tween 80 + 50% 0.9% NaCl. DMSO was used in the control configuration. Tumor samples were gathered for mass measurement following post‐euthanasia to assess the experimental results.

In order to investigate the relationship of SEPHS2 knockdown with tumor metastasis, DU145 cells stably expressing shSEPHS2 or shNC were harvested, washed and resuspended in a 1:1 mixture of PBS and Matrigel at a concentration of 1 × 10^6^ cells/10 µL. Male BALB/c nude mice (8 weeks old, *n* = 5 per group) were anesthetized with 1% pentobarbital sodium, and a lower midline abdominal incision was made to expose the urogenital tract. Ten microliters of the cell suspension was slowly injected into the left ventral lobe of the prostate using a 30‐gauge Hamilton microsyringe. The needle was retained for 30 s post‐injection to prevent reflux, and the abdominal wall and skin were closed with absorbable sutures. Mice were housed under standard conditions without any drug intervention. At 6 weeks post‐implantation, mice were euthanized, and the primary tumors, livers, and lungs were carefully dissected. The liver surface was examined macroscopically for metastatic nodules, and the number of visible nodules was counted.

### 4.16 Immunohistochemistry (IHC) Assay

Tumor tissue processing, including fixation, paraffin‐embedding, and sectioning, was performed by Runnerbio Biotech Co., Ltd. (Shanghai, China). For H&E and IHC staining, the tissue sections were deparaffinized and rehydrated. After antigen repairment, the endogenous peroxidase enzyme was quenched by 3% H_2_O_2_ in methanol. Then the sections were blocked in 10% donkey serum for 1 h and incubated with primary antibody at 4°C overnight. After being cleaned with PBS the next day, the samples were incubated with secondary antibodies tagged with peroxidase for 1 h. This was followed by the addition of DAB (Beyotime, P0202) and hematoxylin counterstaining (Beyotime, C0107). The sections were scanned and analyzed under the Leica Aperio VERSA Slide Scanner.

### 4.17 Transmission Electron Microscopy (TEM)

DU145 cells (shNC and shATF4) were treated with RSL3 (4 µM) for 4 h, harvested by centrifugation, and fixed with 2.5% glutaraldehyde at 4°C for 2–4 h. All subsequent procedures, including postfixation with 1% osmium tetroxide, dehydration, Epon 812 resin infiltration and polymerization (60°C, 48 h), ultrathin sectioning (60–80 nm), staining with 2% uranyl acetate and lead citrate, and imaging, were performed by Wuhan Hela Biotechnology Co., Ltd. (Wuhan, China) using a transmission electron microscope (FEI Tecnai G2 20 Twin, 200 kV). Representative images were captured at various magnifications. Scale bars are indicated in the corresponding images.

### 4.18 Utilization and Analysis of Public Databases

Gene expression data comparing experimental and control groups in several cancer cell lines were downloaded from the Gene Expression Omnibus (GEO) database. The Tidyverse software package was used in R to analyze the data.

The HIPLOT online platform [45] (https://hiplot.com.cn/home/index.html) was used to analyze the DEGs.

### 4.19 Statistical Analysis

Unless otherwise noted, experimental results are reported as mean and standard deviation (mean ± SD). Statistical analyses were performed using GraphPad Prism 9.5.0. Unpaired two‐tailed Student's *t*‐tests assessed differences between two groups. One‐way ANOVA (multiple groups) or two‐way ANOVA (two independent factors) with appropriate post hoc tests were used for complex comparisons. A *p*‐value threshold of < 0.05 defined statistical significance, with asterisks in figures denoting: *p < 0.05, **p < 0.01, and ***p < 0.001; ns indicates non‐significance.

## Author Contributions

S.B., X.D., and Z.T. contributed equally to this work. Z.T., H.X., Y.X.F., and J.S. designed the study. S.B., Z.T., H.X., J.Z., and Z.W. performed all the experiments and data analysis. Z.T., Y.X.F., and J.S. contributed to original manuscript drafting. S.B., H.X., X.D., WQ.G., B.D, and J.S. helped in manuscript editing. All authors read and approved the final manuscript.

## Funding

This work was supported by the National Natural Science Foundation of China (82372698 to B.D.), the Beijing Life Oasis Foundation (CT202501070050 to J.S.), the Renji Hospital PI Leadership Award Project (RJTJ25‐MS‐042 to J.S.), and the Shanghai Yangtze River Delta Medical Innovation Life and Health Industry Foundation (2026‐YRDFHI‐063 to J.S.).

## Ethics Statement

This research was carried out in compliance with both national and international norms of ethical conduct in animal experimentation, adhering to the ethical codes outlined by the Ren Ji Hospital's Ethical Review Committee for Animal Experimentation (approval number: RA‐2022‐191).

## Conflicts of Interest

The authors declare no conflicts of interest.

## Supporting information




**Supporting File 1**: mco270908‐sup‐0001‐SuppMat.docx

## Data Availability

The RNA‐seq dataset from DU145 cells used in this study was generated in our previous work [44] and has been deposited in the NCBI Gene Expression Omnibus (GEO) database (https://www.ncbi.nlm.nih.gov/geo/) under accession number GSE282995. Other publicly available datasets analyzed in this study were retrieved from GEO (accession numbers: 232034, 236253, and 207741). Additional experimental data generated during the current study are available from the corresponding author upon reasonable request.
