# Supplementary material for: Elevated Expression of Selenoprotein Synthesis Enzyme SEPHS2 Driven by ATF4 Promotes the Resistance to Ferroptosis in Prostate Cancer
Source: MedComm (2020). 2026 Aug 9;7(8):e70908. doi: 10.1002/mco2.70908 (PMC13454560; doi:10.1002/mco2.70908)
Supplement: Supplementary file 1 [file MCO2-7-e70908-s001.docx]

Elevated expression of selenoprotein synthesis enzyme SEPHS2 driven by ATF4 promotes the resistance to ferroptosis in prostate cancer

Shijia Bao^1, #^, Xinxing Du^2, #^, Zhenkeke Tao^1, #^, Huixiang Xiao^3^, Jia Zhang^1^, Zhenzhen Wen^1^, Wei-Qiang Gao ^1,4,5^, Baijun Dong^2^, Yu-Xiang Fang^1, *^, Jianjun Sha^2, *^.

Affiliations:

1. State Key Laboratory of Systems Medicine for Cancer, Ren Ji Hospital, Shanghai Cancer Institute, Shanghai Jiao Tong University School of Medicine, Shanghai, China.

2. Department of Urology, Ren Ji Hospital, School of Medicine, Shanghai Jiao Tong University, Shanghai, China.

3. Department of Urology, Shanghai General Hospital, Shanghai Jiao Tong University School of Medicine, Shanghai, China.

4. School of Biomedical Engineering & Med-X Research Institute, Shanghai Jiao Tong University, Shanghai, China

5. Shanghai Key Laboratory of Cancer System Regulation and Clinical Translation, Shanghai, China

#: These authors contribute equally.

*: Corresponding authors: Yu-Xiang Fang (Rm 1515, Bldg 5, 160 Pujian Road, Shanghai 200127, China. Tel: 86-21-68382511, Email: [fyx2003@sjtu.edu.cn](mailto:fyx2003@sjtu.edu.cn)). Jianjun Sha (Rm 701, Bldg 1, 145 Shandong Zhong Road, Shanghai 200001, China. Tel: 86-21-53882055, Email: [shajianjunrj@126.com](mailto:shajianjunrj@126.com)).

Supplementary Figures and Legends

**Figure S1 Ferroptosis inducers trigger SEPHS2 upregulation.**

**(A)** Schematic representation of the Selenium synthesis metabolic pathways. **(B)** SEPHS2 expression was upregulated after treatment with ferroptosis inducers in several datasets. **(C)** Viability of prostate cancer cells treated with Erastin. **(D)** The expression of SEPHS2 in different prostate cancer cell lines. **(E)** Western blot analysis of SEPHS2 expression in LNCaP cells after treatment with RSL3 or Erastin for 0–72 h. **(F)** Western blot analysis of SEPHS2 expression in LNCaP cells treated for 72 h with Erastin **±** Fer-1 or 3-methyladenine (3-MA). Values represent the mean ± SD (*n* = 3 per group). Statistical analysis was performed using unpaired two-tailed Student’s t-test in (B), and two-way ANOVA followed by Tukey’s test in (C).

**Figure S2 SEPHS2 overexpression promotes tumor cell proliferation, migration and invasion.**

**(A)** SEPHS2 overexpression in LNCaP and C4-2B (Vector vs. SEPHS2-OE) cells, as validated by Western blot. **(B)** SEPHS2 knockdown in DU145 and PC-3 (shNC vs. shSEPHS2) cells, as validated by Western blot. **(C)** Proliferative capacity of C4-2B (Vector vs. SEPHS2-OE). **(D)** Representative micrographs and quantification of C4-2B (Vector vs. SEPHS2-OE) cell migration and invasion. Scale bar: 50 μm. Values represent the mean ± SD (*n* = 3 per group). Statistical analysis was performed using two-way ANOVA and unpaired two-tailed Student’s t-test in (C), and unpaired two-tailed Student’s t-test in (D).

**Figure S3 SEPHS2 deficiency attenuates tumor cell proliferation, migration and invasion.**

**(A)** Proliferative capacity of PC-3 (shNC vs. shSEPHS2) cells (*n* = 3 per group). **(B)** Representative micrographs and quantification of PC-3 (shNC vs. shSEPHS2) cell migration and invasion (*n* = 3 per group). Scale bar: 50 μm. **(C)** Representative gross images of orthotopic tumors and liver metastatic nodules (arrows) in DU145 (shNC and shSEPHS2) xenografts. Bilateral spermatic cords were preserved for anatomical orientation. **(D)** Quantification of liver metastatic nodules per mouse in the indicated groups (*n* = 5 per group). Values represent the mean ± SD. Statistical analysis was performed using two-way ANOVA with Tukey’s test and one-way ANOVA with Dunnett’s test in (A), and one-way ANOVA with Dunnett’s test in (B) and (D).

**Figure S4 Neither overexpression nor knockdown of SEPHS2 affects basal apoptosis in tumor cells.**

**(A)** Basal apoptosis levels in LNCaP and C4-2B (Vector vs. SEPHS2-OE) cells. **(B)** Basal apoptosis levels in DU145 and PC-3 (shNC vs. shSEPHS2) cells. Values represent the mean ± SD (*n* = 3 per group). Statistical significance was analyzed by unpaired two-tailed Student’s t-test in (A) and one-way ANOVA with Dunnett’s test in (B).

**Figure S5 Knockdown of SEPHS2 enhances the sensitivity of tumor cells to ferroptosis.**

**(A)** Cell viability of C4-2B (Vector vs. SEPHS2-OE) and PC-3 (shNC vs. shSEPHS2) cells treated for 24 h with RSL3, Erastin, or RSL3/Erastin + Fer-1. **(B)** Lipid ROS levels in C4-2B (Vector vs. SEPHS2-OE) and PC-3 (shNC vs. shSEPHS2) cells treated for 24 h with RSL3 or Erastin. **(C)** Expression of selenoproteins in LNCaP and C4-2B cells with SEPHS2 overexpression. **(D)** Rescue effect of sodium selenite (Na_2_SeO_3_) on ferroptosis sensitivity in DU145 and PC-3 (shNC and shSEPHS2) cells treated with vehicle (sterile water) or Na_2_SeO_3_ (0.1 μM) in the presence of RSL3 or Erastin for 24 h. **(E)** Western blot analysis of TXNRD1 and GPX4 expression in DU145 shSEPHS2 cells treated with vehicle or sodium selenite (Na_2_SeO_3_, 0.1 μM) for 24 h. Values represent the mean ± SD (*n* = 3 per group). Statistical significance was analyzed by unpaired two-tailed Student’s t-test for C4-2B cells and one-way ANOVA with Dunnett’s test for PC-3 cells in (A–B), and by one-way ANOVA with Dunnett’s test in (D).

**Figure S6 Ferroptosis inducers upregulate ATF4 expression.**

**(A)** The heatmap showing expression change of ferroptosis-related transcription factors after RSL3 treatment in DU145 cells. **(B)** Western blot analysis of ATF4 expression in LNCaP cells following RSL3 treatment with or without Fer-1.

**Figure S7 ATF4 modulates ferroptosis resistance in tumor cells.**

**(A)** Western blot analysis of phosphorylated eIF2α (p-eIF2α) and total eIF2α in LNCaP cells treated with or without RSL3 for 24 h. GAPDH served as a loading control. **(B)** ATF4 overexpression in LNCaP cells, and knockdown in DU145 and PC-3 cells, as confirmed by Western blot and qRT-PCR. **(C)** Western blot and qRT-PCR analysis of SEPHS2 expression in ATF4-knockdown PC-3 cells. **(D)** Cell viability of PC-3 (shNC vs. shATF4) cells treated for 24 h with RSL3, Erastin, or RSL3/Erastin + Fer-1. **(E)** The expression of selenoproteins after knocking down ATF4 in PC-3 cells. **(F)** Representative transmission electron microscopy (TEM) images of DU145 (shNC and shATF4) cells treated with RSL3 for 4 h. Overview of cellular ultrastructure (left column), mitochondrial morphology (middle column), and the high-magnification image of mitochondrial ultrastructure (right column) was observed by TEM, as indicated by red arrows. Scale bars: 2 μm (left column), 1 μm (middle column), and 500 nm (right column). **(G)** Immunoblot analysis of p62, LC3B-I/II, and TOM20 in DU145 (shNC and shATF4) cells treated with RSL3 for 0, 4, and 8 h. Values represent the mean ± SD (*n* = 3 per group). Statistical significance was analyzed by unpaired two-tailed Student’s t-test in (B), and one-way ANOVA with Dunnett’s test in (B-D).

**Figure S8 SEPHS2 overexpression rescues ATF4 knockdown-induced ferroptosis sensitivity.**

**(A)** Assessment of cell viability in indicated cells following 24 h exposure to RSL3 or Erastin. **(B)** Lipid ROS levels in indicated cells after 24-hour treatment with RSL3. **(C)** The expression of selenoproteins in indicated cells. Statistical significance was analyzed by one-way ANOVA with Dunnett’s test.

| **Supplementary Tables**  **Table S1: Primers for plasmid construction** | | |
| --- | --- | --- |
| Plasmid | Forward | Reverse |
| shSEPHS2 #1 | 5’- caccg TGGTAGAAGATCCCTACATGACTCGAG TCATGTAGGGATCTTCTACCA tttttg -3’ | 5’- aaac caaaaa TGGTAGAAGATCCCTACATGACTCGAG TCATGTAGGGATCTTCTACCA c -3’ |
| shSEPHS2 #2 | 5’- caccg TTGTTGATAAATCTAAATTGT CTCGAG ACAATTTAGATTTATCAACAA tttttg -3’ | 5’- aaac caaaaa TTGTTGATAAATCTAAATTGTCTCGAG ACAATTTAGATTTATCAACAA c -3’ |
| shATF4 #1 | 5’- caccg GAATGATCTGGAGTGGAGGACCTCGAG GTCCTCCACTCCAGATCATTC tttttg -3’ | 5’- aaac caaaaa GAATGATCTGGAGTGGAGGACCTCGAG GTCCTCCACTCCAGATCATTC c -3’ |
| shATF4 #2 | 5’- caccg CTATACCCAACAGGGCATCCACTCGAG TGGATGCCCTGTTGGGTATAG tttttg -3’ | 5’- aaac caaaaa CTATACCCAACAGGGCATCCACTCGAG TGGATGCCCTGTTGGGTATAG c -3’ |
| oeATF4 | 5’- ccggaattcATGACCGAAATGAGCTTCCTGAGCAGCGAGGTGTTGGTGG -3’ | 5’- atagtttagcggccgcCTAAGCGTAATCTGGTACGTCGTATGGGTACTTGTCATCGTCGTCC -3’ |
| oeSEPHS2 | 5’- CCGCTCGAGGCCACCATGGCGGAAGCCTCGGCGACGGGC -3’ | 5’- CCGGAATTCCGAGCTAGGCTCAGAGGAGGCATT -3’ |
| mut-oeSEPHS2 | 5’- TCCGGCATGAAGGGCTGCGGCTGCAAGGTCCCGCA -3’ | 5’- TGCGGGACCTTGCAGCCGCAGCCCTTCATGCCGGA -3’ |

| **Table S2: qPCR primers** | | |
| --- | --- | --- |
| Gene | Forward | Reverse |
| ATF4 | TTCTCCAGCGACAAGGCTAAGG | CTCCAACATCCAATCTGTCCCG |
| SEPHS2 | TGAGGAGGAACGCGAAAAGG | CCAAGGGTTGACCACCGTTT |
| GAPDH | GTCTCCTCTGACTTCAACAGCG | ACCACCCTGTTGCTGTAGCCAA |
| ChIP-SEPHS2-BS1 | TGCCGATCATGAGGTTTGC | AACGTCACGTGCTTTCCTTCG |
| ChIP-SEPHS2-BS2 | TGGCGCGGACGCAGCTCACT | AAAAACAGAAAGCATGAGAA |
| ChIP-SEPHS2-BS3 | GCACTTTGGGAGGCCGAGG | CTCCTGAATAGCTGGGATTA |
| ChIP-SEPHS2-BS4 | AGATTGTGCATGCCGTGG | TCCGCCATGGCGCCTGCCCG |
| ChIP-SEPHS2-BS5 | GAGAGCGAAGGAAAGCACGT | TTATAGCCCTGGCGATTGAC |
| ChIP-SEPHS2-BS6 | TTATTTACAGAGATATAGGAATTTTAGAAT | ACTGGAAGTCCAGACACCTG |

| **Table S3: Antibodies** | | | | |
| --- | --- | --- | --- | --- |
| Antibody | Catalog No. | Company | Dilution factor for WB | Dilution factor for IHC |
| SEPHS2 | GT39876 | Sigma | 1:1000 | 1:100 |
| ATF4 | 11815S | CST | 1:1000 | 1:100 |
| TXNRD1 | A16631 | Abclonal | 1:1000 | - |
| TXNRD2 | A5490 | Abclonal | 1:1000 | - |
| SEPHS1 | A19919 | Abclonal | 1:1000 | - |
| GPX1 | A1110 | Abclonal | 1:1000 | - |
| GPX4 | A21440 | Abclonal | 1:1000 | - |
| VIMP | A15013 | Abclonal | 1:1000 | - |
| HRP-GAPDH | HRP-60004 | Proteintech | 1:2000 | - |
| Anti-rabbit IgG | 7074S | CST | 1:5000 | - |
| Anti-mouse IgG | 7076S | CST | 1:5000 | - |
| 4-HNE | ab48506 | Abcam | 1:100 | 1:100 |
| KI67 | ab15580 | Abcam | 1:100 | 1:100 |

| **Table S4: Venn summary of ferroptosis-induced DEGs (up/down) across GSE datasets** | | | | | | | | |
| --- | --- | --- | --- | --- | --- | --- | --- | --- |
| up-regulated | | |  | | down-regulated | | | |
| HMOX1 | BTN2A1 |  | | E2F8 | PCNA | FAM72D | CENPA | HJURP |
| GABARAPL1 | RDH10 |  | | BUB1B | OIP5 | DSN1 | CCDC57 | SUV39H1 |
| DNAJB2 | RTCA |  | | TOP2A | SLC29A1 | ECT2 | CRACR2B | TFDP1 |
| TAX1BP1 | CLIP4 |  | | TUBB | KIF4A | CSE1L | SKA3 | POLD3 |
| CYB5R1 | VPS26A |  | | LIG1 | UBE2C | TACC3 | FBXO5 | TMEM107 |
| IRF7 | TOR1AIP2 |  | | CCNA2 | AURKA | ASF1B | HADH | LIN9 |
| PPP3CC | VRK3 |  | | CDCA7 | ZWINT | ANP32A | ARHGAP11A | MNS1 |
| OSTM1 | SLC30A1 |  | | NUCKS1 | MCM4 | ISYNA1 | RBMX | CDC6 |
| PSAP | RABGEF1 |  | | MCM7 | CDCA8 | HDGF | DIAPH3 | RFC2 |
| UBE2A | FTH1 |  | | POLD1 | GINS1 | CDK1 | FDPS | PRIMPOL |
| MAP1LC3B | NFE2L2 |  | | CENPI | POLA1 | NUP93 | BZW2 | TFAP4 |
| NQO1 | NDEL1 |  | | MCM2 | TK1 | TMEM123 | ATAD3A | WDR54 |
| OSGIN1 | SAV1 |  | | TONSL | CDCA5 | MELK | SHMT1 | KNSTRN |
| SCPEP1 | CASP4 |  | | CDCA3 | CDK2 | RAD54L | SEPHS1 | LSM2 |
| SQSTM1 | CNST |  | | MCM3 | ORC1 | CHAF1A | NRM | HAUS4 |
| EIF1B | OTUD1 |  | | TCF19 | SSBP4 | RRM1 | NUDT1 | ZMYND10 |
| TOM1 |  |  | | RECQL4 | TPX2 | CHEK1 | CTSV | GAMT |
| TRIM16 |  |  | | POLE2 | NDC80 | CDC45 | SNHG4 | CCDC15 |
| IGFBP3 |  |  | | UHRF1 | PHF19 | CCDC138 | MCMBP | WWTR1 |
| ARRDC4 |  |  | | MTHFD1 | TRAP1 | BLM | SPC25 | SFXN4 |
| RIT1 |  |  | | KIF15 | NEIL3 | MIPEP | HCFC1R1 | ACD |
| BRF2 |  |  | | CDC20 | DEPDC1B | PBK | EMP2 | PDIA5 |
| MT1F |  |  | | MCM6 | CHAF1B | TTK | RCCD1 | EXOSC5 |
| UPP1 |  |  | | ALDH1B1 | FANCE | RMI2 | CTSC | RGS19 |
| RNF11 |  |  | | ARHGEF39 | NDC1 | PARPBP | PRPS2 | RRM2 |
| CD55 |  |  | | SAPCD2 | TYMS | PDIA6 | PKMYT1 | E2F1 |
| HSPB8 |  |  | | ATAD2 | SSRP1 | KPNA2 | CENPK | CENPQ |
| OPTN |  |  | | MSH2 | WDHD1 | B4GALT2 | NUP35 | FANCB |
| DUSP10 |  |  | | KIF23 | NCAPH | GRK6 | SLC25A5 | DEK |
| TIPARP |  |  | | LMNB1 | TMEM109 | XRCC2 | BRCA1 | DEPDC1 |
| SEPHS2 |  |  | | PLK1 | CTPS2 | NUP85 | POLD2 | HMGB1 |
| TERF2IP |  |  | | DLGAP5 | TRIP13 | SLC25A10 | STIL | RBBP7 |
| RNF19B |  |  | | SHCBP1 | KIF22 | TMPO | IVNS1ABP | SNRPA |
| YPEL5 |  |  | | KIF11 | TERT | KIF2C | MND1 | CENPM |
| ATP6V0D1 |  |  | | WDR76 | BRIP1 | CENPU | ESCO2 | AGPAT5 |
| PNPLA8 |  |  | | CCNB2 | SKP2 | XRCC5 | DPF1 | TRAIP |
| ABI1 |  |  | | RFT1 | SFXN2 | REEP4 | SMC3 | FAM72B |
| P4HA1 |  |  | | TMPO-AS1 | NT5DC2 | VRK1 | CEP55 | PAICS |
| GCLM |  |  | | KIFC1 | NUF2 | ISOC2 | EME1 | RAD51AP1 |
| MIR22HG |  |  | | CDCA2 | RFC4 | POC1A | FAM72C | PPP2R3B |
| IL11 |  |  | | PSRC1 | DSCC1 | SPDL1 | PSIP1 | RNPS1 |
| ORAI3 |  |  | | FEN1 | IDH2 | C9orf40 | RTKN2 |  |
| GNPDA1 |  |  | | FAM83D | CCDC34 | PRR7 | AKAP7 |  |
| TXNRD1 |  |  | | FANCI | FANCG | MCM10 | FAM111B |  |
| AUP1 |  |  | | FANCD2 | GINS2 | ZNF367 | SLC19A1 |  |
| RRAGB |  |  | | HELLS | POLA2 | SPC24 | ARRB2 |  |
| SERINC1 |  |  | | MYBL2 | C4orf46 | MAD2L1 | ITGB3BP |  |
| PANX2 |  |  | | NCAPG | HMMR | PACSIN3 | HAUS8 |  |
| STX3 |  |  | | PDIA4 | AURKB | LRRC45 | GMPPB |  |
| DNAJB1 |  |  | | EXO1 | RFC5 | AK2 | SORD |  |
| DUSP14 |  |  | | SLC39A10 | MCM5 | CENPH | GGCT |  |
| RAB22A |  |  | | CDC7 | RANBP1 | FARSB | DCK |  |
| MOAP1 |  |  | | BIRC5 | RFC3 | NEK2 | CDT1 |  |

| Table S5: KEGG enrichment of ferroptosis-associated up-regulated consensus genes | | | | |
| --- | --- | --- | --- | --- |
| Description | Count | GeneRatio | qvalue | geneID |
| Mitophagy - animal | 5 | 0.135 | 0.0012 | MAP1LC3B/GABARAPL1/OPTN/TAX1BP1/SQSTM1 |
| Ferroptosis | 4 | 0.108 | 0.0012 | MAP1LC3B/HMOX1/GCLM/  FTH1 |
| Mineral absorption | 4 | 0.108 | 0.0037 | SLC30A1/MT1F/HMOX1/FTH1 |
| Selenocompound  metabolism | 2 | 0.054 | 0.0466 | SEPHS2/TXNRD1 |
| Fluid shear stress  and atherosclerosis | 4 | 0.108 | 0.0466 | SQSTM1/NFE2L2/HMOX1/NQO1 |
| Autophagy - animal | 4 | 0.108 | 0.0466 | MAP1LC3B/GABARAPL1/  RRAGB/SQSTM1 |
| Hepatocellular  carcinoma | 4 | 0.108 | 0.0746 | TXNRD1/NFE2L2/HMOX1/NQO1 |
| Shigellosis | 4 | 0.108 | 0.2410 | MAP1LC3B/RRAGB/SQSTM1/  CASP4 |
| Cellular senescence | 3 | 0.081 | 0.2926 | SQSTM1/PPP3CC/IGFBP3 |
| Protein processing  in endoplasmic  reticulum | 3 | 0.081 | 0.3150 | NFE2L2/DNAJB1/DNAJB2 |
| Synaptic vesicle  cycle | 2 | 0.054 | 0.3150 | ATP6V0D1/STX3 |
| Ubiquinone and  other terpenoid-quinone biosynthesis | 1 | 0.027 | 0.3150 | NQO1 |
| NOD-like receptor  signaling pathway | 3 | 0.081 | 0.3150 | MAP1LC3B/GABARAPL1/CASP4 |

| **Table S6: Ferroptosis-related transcription factors (RNA-seq)** | | | | | | | | | | |
| --- | --- | --- | --- | --- | --- | --- | --- | --- | --- | --- |
| Gene | DMSO-1 | DMSO-2 | DMSO-3 | RSL3-1 | RSL3-2 | RSL3-3 | log2(Fold_change) | p-value | q-value | Regulation |
| TP53 | 2608.351 | 3223.149 | 3458.366 | 5026.875 | 6223.004 | 6099.392 | 0.901439 | 1.59E-08 | 7.29E-07 | up |
| ATF4 | 8084.068 | 8434.325 | 8218.355 | 11562.33 | 11180.86 | 11556.87 | 0.471604 | 8.47E-08 | 3.19E-06 | up |
| EPAS1 | 1549.19 | 1633.086 | 1683.611 | 1891.433 | 2061.99 | 1852.692 | 0.255231 | 0.021922 | 0.075758 | up |
| SP1 | 1007.358 | 952.7829 | 916.5642 | 780.2292 | 722.7716 | 730.5378 | -0.36542 | 0.001846 | 0.012061 | down |
| YAP1 | 1125.665 | 1140.113 | 1053.793 | 901.6212 | 755.0275 | 770.0587 | -0.45148 | 0.000521 | 0.004521 | down |
| WWTR1 | 961.8557 | 773.5199 | 731.2031 | 712.7891 | 561.492 | 487.4244 | -0.48557 | 0.011452 | 0.047191 | down |
| NFE2L2 | 329.7191 | 216.0119 | 210.9634 | 203.3576 | 146.9436 | 108.9819 | -0.72034 | 0.012056 | 0.048947 | down |
| BACH1 | 163.1095 | 86.04625 | 81.92752 | 58.10217 | 33.45059 | 32.33528 | -1.41738 | 0.000245 | 0.002509 | down |
| HIF1A | 2115.523 | 1284.42 | 1365.117 | 819.6556 | 481.4495 | 440.7179 | -1.45139 | 1.60E-07 | 5.68E-06 | down |
